# Vascular limitations in blood pressure regulation with age in women: Insights from exercise and acute cardioselective β‐blockade

**DOI:** 10.1113/EP091843

**Published:** 2024-10-04

**Authors:** Matthew J. Studinski, Christine Bowlus, James A. Pawelczyk, Jocelyn M. Delgado Spicuzza, Jigar Gosalia, Swapan Mookerjee, Matthew D. Muller, Jason Fragin, David N. Proctor

**Affiliations:** ^1^ Integrative and Biomedical Physiology, Intercollege Graduate Degree Program The Pennsylvania State University University Park Pennsylvania USA; ^2^ Department of Kinesiology The Pennsylvania State University University Park Pennsylvania USA; ^3^ Department of Health and Exercise Science Commonwealth University of Pennsylvania Bloomsburg Pennsylvania USA; ^4^ School of Medicine Case Western Reserve University Cleveland Ohio USA; ^5^ Penn State Heart and Vascular Institute Penn State College of Medicine Hershey Pennsylvania USA

**Keywords:** ageing, cardiovascular, esmolol, exercise haemodynamics, women

## Abstract

Younger women rely on altering cardiac output ($\dot{Q}$) to regulate blood pressure (BP). In contrast, older women rely more on altering vascular tone. However, evidence suggests that the ability to alter systemic vascular conductance (SVC) is diminished in older women. In the present study, cardioselective β‐blockade was utilized to diminish the relative contribution of $\dot{Q}$ to BP regulation and thereby evaluate age‐related vascular limitations in women at rest and during large muscle dynamic exercise. Younger (*n* = 13, mean age 26.0 years) and older (*n* = 14, mean age 61.8 years) healthy women performed submaximal bouts of semi‐recumbent cycling exercise at varying intensities while receiving an intravenous infusion of esmolol, a β_1_‐antagonist, or saline control in a repeated‐measures crossover design. $\dot{Q}$ was attenuated during esmolol infusion, with greater reductions during exercise (moderate, –1.0 (95% CI, –1.6 to –0.5) L/min, *P *< 0.001; heavy, –2.0 (95% CI, –2.6 to –1.5) L/min, *P *< 0.001) than seated rest (–0.5 (95% CI, –1.1 to 0.0) L/min, *P* = 0.048), and this reduction was not significantly different between age groups (*P* = 0.122). Older women exhibited a greater attenuation in mean arterial pressure (MAP) during esmolol (–7 (95% CI, –9 to –4) mmHg, *P *< 0.001) relative to younger women (–2 (95% CI, –5 to 0) mmHg, *P* = 0.071). These changes coincided with a greater reduction of SVC in the younger women during esmolol (–15 (95% CI, –20 to –10) mL/min/mmHg, *P *< 0.001) compared to older women (–3 (95% CI, –9 to 2) mL/min/mmHg, *P* = 0.242). Together, these findings provide evidence that older, postmenopausal women have a diminished ability to adjust SVC in order to regulate MAP.

## Abstract (highlights)

- **What is the central question of this study?**
  Will older women have a greater reduction in mean arterial pressure during cardioselective β‐blockade compared to younger women? And will older women exhibit a smaller reduction in systemic vascular conductance in response to cardioselective β‐blockade relative to younger women?
- **What is the main finding and its importance?**
  Cardioselective β‐blockade revealed a limitation in the systemic vasoconstrictor capacity of older women to maintain blood pressure both at rest and during dynamic exercise. These findings may have significant clinical implications, particularly during hypotensive stressors such as heat stress, hypovolemia and orthostatic hypotension.

## 1 INTRODUCTION

The principal determinants of mean arterial pressure (MAP) are cardiac output ($\dot{Q}$) and systemic vascular conductance (SVC), as defined by Ohm's law:

$$\mathrm{MAP}\frac{\dot{Q}}{\mathrm{SVC}}$$

Ageing has been observed to alter the relative contribution of these two factors to the blood pressure (BP) response. The role of $\dot{Q}$ has been shown to diminish with age (Ogawa et al., 1992; Seffrin et al., 2023; Stratton et al., 1994). Meanwhile, the relative importance of SVC increases with age (Dinenno et al., 2001; Gerhard et al., 1996; Hearon et al., 2020; Koch et al., 2003; Singh et al., 2002).

However, these age‐related changes to the regulation of arterial pressure appear to be sex‐dependent (Baker et al., 2018; Hanson et al., 2021; Ji et al., 2020; Joyner et al., 2016; Proctor & Parker, 2006; Trinity et al., 2018), with women experiencing greater increases in both resting and exercise BP with age (Martin et al., 1991; Narkiewicz et al., 2005; Ogawa et al., 1992; Proctor et al., 2003). Younger women appear to rely on increasing heart rate (HR), and consequently their $\dot{Q}$, when experiencing a drop in BP (Credeur et al., 2014; Kim et al., 2011). Older women, on the other hand, demonstrate an increased reliance on lowering SVC in response to a hypotensive stimulus (Credeur et al., 2014). Despite an increased reliance on altering vascular tone, evidence suggests that older women have a diminished ability to adequately adjust SVC to acute changes in arterial pressure (Credeur et al., 2014; Trinity et al., 2018). It has been hypothesized that this transition to a dependence on vascular responses to changes in BP is likely mediated by both reduced cardiac responsiveness and vascular changes in older women (Fisher et al., 2009, 2007; Hart et al., 2011).

The complex interplay between these myriad changes is perhaps best examined during exercise, which relies on coordinated changes to both central (HR, $\dot{Q}$) and peripheral vascular responses (vasodilatation and vasoconstriction) to maintain systemic BP and meet metabolic demands. The majority of previous work examining differences in exercise BP regulation with age in women has focused on small muscle exercise (Choi et al., 2012; Fadel et al., 2004; Grotle et al., 2023; Kang et al., 2022; Petrofsky & Lind, 1975; Ranadive et al., 2017; Trinity et al., 2018; Watso et al., 2020; Wenner et al., 2022). This is important to note, as the relative contributions of central and peripheral vascular responses differ between large and small muscle mass exercises (Pawelczyk et al., 1997). While small muscle mass exercise is ideal for analysing oxidative capacity, large muscle mass exercise is better suited to examine the integration of central and peripheral limitations (Poole & Richardson, 1997). Therefore, these previous observations may not be reflective of the BP response under conditions involving large muscle mass exercise, warranting further examination.

In the present study, we used cardioselective β‐blockade to elicit a greater vasoconstrictor response to exercise in order to unmask the limits of systemic vasoconstriction in older women (Pawelczyk et al., 1997). β_1_‐Blockade attenuates sympathetically mediated increases in contractility and HR. This results in a reduced ability to increase $\dot{Q}$ and instigates a greater reliance on altering vascular tone to maintain MAP. This uncoupling of the central and peripheral vascular responses to exercise allows us to test the limits of the vasculature to maintain BP. The central questions of this study were accordingly, (1) Will older women have a greater reduction in MAP during cardioselective β‐blockade compared to younger women? and (2) Will older women exhibit a smaller reduction in SVC in response to cardioselective β‐blockade relative to younger women? We hypothesized that older women would demonstrate a reduced ability, relative to younger women, to compensate for an experimentally induced reduction in $\dot{Q}$ during exercise, resulting in a greater attenuation of MAP and a smaller reduction in SVC.

## 2 METHODS

### 2.1 Ethics approval

All protocols were approved by the Institutional Review Board of Penn State University (Study Number 00010736) in accordance with the *Declaration of Helsinki*. Each subject voluntarily gave their written, informed consent prior to participation in this study.

### 2.2 Subject characteristics

Subjects were healthy women between 20 and 32 (younger group) or 55 and 70 (older group) years of age. Subjects who were screened into this protocol were non‐smokers and were not taking any medications affecting heart rate or contractility. Exclusion criteria included: pregnant and nursing women, those with a history of cardiovascular, pulmonary, renal, liver or thyroid disease, uncontrolled diabetes, uncontrolled hypertension, and recreational drug use. Physical characteristics of the 27 (13 younger, 14 older) women who completed both experimental visits are provided in Table 1.

**TABLE 1:** Subject characteristics.

	Younger (18–35)	Older (55–70)	*P*
Number	13	14	
Anthropometrics			
Age (years)	26 ± 4	62 ± 4	<0.001
Height (cm)	164 ± 11	163 ± 5	0.746
Weight (kg)	70 ± 13	68 ± 9	0.682
Waist circumference (cm)	79 ± 8	84 ± 10	0.188
Leg lean mass (kg)	13.9 ± 2.7	11.4 ± 1.4	0.007
Cardiovascular			
SBP (mmHg)	107 ± 6	117 ± 11	0.010
DBP (mmHg)	64 ± 5	64 ± 6	0.993
MAP (mmHg)	78 ± 5	82 ± 7	0.134
Resting HR (beats/min)	71 ± 13	66 ± 9	0.281
Haemoglobin (g/dL)	13.7 ± 0.8	13.4 ± 0.7	0.374
Activity and fitness			
Total activity (MET min/week)	4271 ± 3063	2476 ± 1253	0.093
${\dot{V}}_{O_{2}\mathrm{peak}}$ (mL/min)	2437 ± 605	1586 ± 310	<0.001
${\dot{V}}_{O_{2}\mathrm{peak}}$/LLM (mL/min/kg)	176 ± 34	140 ± 23	0.004
Moderate workload (W)	81 ± 21	46 ± 9	<0.001
Heavy workload (W)	117 ± 31	72 ± 15	<0.001

*Note*: All values are presented as means ± SD. Significance was assessed using unpaired *t*‐tests with α = 0.05. DBP, diastolic blood pressure; LLM, leg lean mass; MAP, mean arterial pressure; MET, metabolic equivalent; SBP, systolic blood pressure; ${\dot{V}}_{O_{2}\mathrm{peak}}$, peak oxygen uptake.

### 2.3 Experimental design

This study was conducted using a randomized, placebo‐controlled, crossover design. Subjects, data collectors and data analysts were blinded to treatment orders, but clinical staff responsible for administering drugs and monitoring participant safety were not blinded to treatment orders.

Patients were asked to complete two visits to the lab, with each visit including exercise on an electronically braked leg cycle ergometer modified to the semi‐recumbent position (Lode, Groningen, The Netherlands) with adjustable arm rests placed at approximately heart level on both sides of the subject. The initial visit consisted of clinical screening tests (medical history, physical exam and blood draw) and a step–ramp–step semi‐recumbent cycling protocol to estimate exercise thresholds (Iannetta et al., 2020). The infusion visit consisted of one bout of progressive, semi‐recumbent cycling while receiving an intravenous infusion of saline and another bout of exercise while receiving an infusion of esmolol separated by 45 min. Subjects were randomly assigned which drug to receive first. Prior to each study visit, subjects were instructed to avoid alcohol, over‐the‐counter cold and flu medications (such as nasal decongestants and non‐steroidal anti‐inflammatory drugs) and strenuous exercise for 24 h, to eat a small meal 2–3 h prior to coming to the laboratory, and to refrain from items containing caffeine for 12 h prior to this visit.

#### 2.3.1 Subject screening

After giving informed consent, subjects were asked to complete a medical history questionnaire and the International Physical Activity Questionnaire—Short Form (Craig et al., 2003). Women of childbearing potential (pre‐menopausal) took a urine pregnancy test to ensure they were not pregnant. Participants then completed a physical exam and a resting electrocardiogram. A venous blood sample was collected by a nurse and sent for analysis (Complete Blood Count and Comprehensive Metabolic Panel; 7.5 mL total). Lab values were verified and signed off by a clinician before infusion began and were obtained no more than 6 weeks prior to the infusion. This information was shared with a clinician who then determined if subjects were eligible for participation in the study.

#### 2.3.2 Determination of exercise intensity domains

**FIGURE 1:**
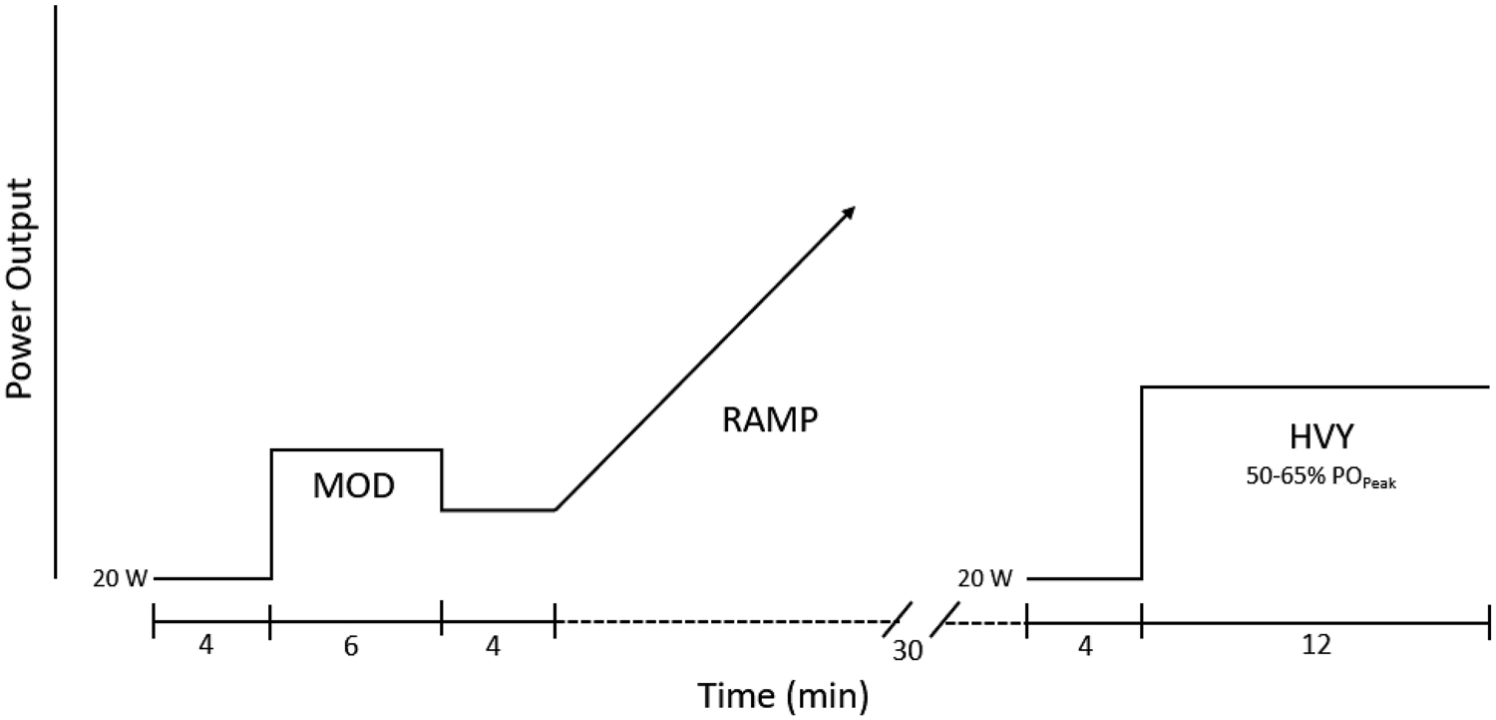
Schematic representation of the step–ramp–step protocol. The step–ramp–step exercise protocol was performed during the initial visit in order to identify exercise intensity thresholds for the subsequent infusion visit. The protocol consists of 4 min at 20 W, 6 min at moderate intensity, 4 min at moderate–easy intensity, and then ramp to exhaustion. After a 30‐min break, 4 min at 20 W, followed by 12 min at heavy intensity. Adapted from Ianetta et al. (2020).

In order to identify exercise intensity thresholds and assign workloads for the infusion visit, subjects completed a step–ramp–step protocol (Figure 1, adapted from Ianetta et al., 2020) during the initial visit. Subjects were asked to complete three exercise bouts on a stationary semi‐recumbent bike. The first bout consisted of very light pedalling for 2 min (20 W) followed by approximately 6 min of pedalling at what should be a moderate effort for the subject.

After a few minutes of rest and/or light pedalling (recovery), the subject was then asked to perform a second bout of exercise, which consisted of a gradual increase in workload (‘ramp’) until the subject could no longer maintain the required pedal speed (50–60 rpm) or needed to stop due to fatigue. The rate of increase in workload was selected on an individual basis such that the ramp protocol lasted between 8 and 12 min.

After 30 min of recovery, the subject pedalled for 2 min at 20 W followed immediately by 12 min of pedalling at 50–65% of peak power output.

Respiratory measurements were collected using a breath‐by‐breath system (Quark CPET, Cosmed, Rome, Italy), which was calibrated prior to each study visit. The respiratory data collected (${\dot{V}}_{O_{2}}$, ${\dot{V}}_{CO_{2}}$, ${\dot{V}}_{E}$, $P_{\mathrm{ETC}O_{2}}$, $P_{\mathrm{ET}O_{2}}$, ${\dot{V}}_{E}$/${\dot{V}}_{CO_{2}}$, ${\dot{V}}_{E}$/${\dot{V}}_{O_{2}}$ and respiratory exchange ratio) was then examined using the exercise thresholds app (https://www.exercisethresholds.com) and intensity thresholds (first and second ventilatory thresholds; VT_1_ and VT_2_, respectively) were determined.

### 2.4 Infusion visit

An intravenous catheter was placed into the subject's non‐dominant arm. Once fully instrumented, a clinician then began administering an infusion of either esmolol or saline (order determined randomly). Subjects received an esmolol loading dose equal to 0.5 mg/kg/fat‐free mass/min administered over the first 3 min (loading dose), followed by a maintenance dose of 0.25 mg/kg/fat‐free mass/min for the remainder of the protocol (maximum of 60 min). Fat‐free mass was determined using a whole‐body dual energy X‐ray absorptiometry scan (Hologic Horizon W DXA system, Hologic, Inc., Marlborough, MA, USA). This dose of esmolol was previously shown by our group to attenuate the HR response to exercise and isoproterenol infusion in younger men without blocking β_2_‐receptors (Muller et al., 2017). Our group also observed that this dose of esmolol did not significantly alter the changes in blood glucose or potassium concentrations relative to saline (Muller et al., 2017). Fat‐free mass, rather than total body mass, was used because fat‐free mass is correlated with total blood volume (Hunt et al., 1998) and esmolol is metabolized by red blood cell esterases (Wiest & Haney, 2012). The volume and rate of saline infusion were matched to that of esmolol.

**FIGURE 2:**
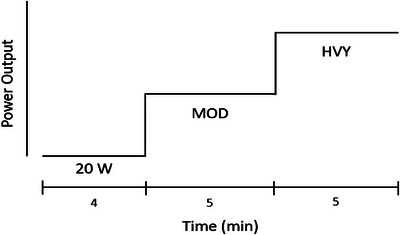
Schematic representation of the infusion study visit protocol. Intravenous infusion began prior to the start of exercise and resting baseline values were collected at least 2 min after the start of the maintenance dose. After baseline measurements were recorded, the semi‐recumbent cycling exercise protocol was performed and consisted of 4 min at 20 W followed by 5 min at moderate intensity (MOD) and last 5 min at heavy intensity (HVY).

Resting baseline values were collected at least 2 min after the start of the maintenance dose. At least 1 min after resting data were collected, subjects began the cycling exercise protocol (Figure 2). Subjects were asked to pedal for 2–4 min at a very light intensity (20 W), followed immediately by 5 min at a moderate intensity (workload intended to elicit 85% of the oxygen consumption observed at the VT_1_), and 5 min of heavy intensity cycling (workload estimated to elicit oxygen consumption halfway between those observed at the VT_1_ and VT_2_). The subjects then rested for 45 min, after which they repeated the exercise protocol while receiving the other treatment. Workloads were assigned based on exercise intensity domains to ensure a similar metabolic milieu between individuals.

HR, $\dot{Q}$, SVC and respiratory gas exchange were recorded electronically and continuously throughout the experiment and are reported as the mean value over the last minute of each stage (baseline, moderate exercise and heavy exercise). HR, $\dot{Q}$ and SVC were collected using impedance cardiography (PhysioFlow Enduro, Manatec Biomedical, Petit Ebersviller, France) and respiratory gas exchange was collected using breath‐by‐breath analysis (Quark CPET, Cosmed). BP was collected at baseline and 3 min into the moderate and heavy exercise stages using an automated sphygmomanometer (Tango M2, SunTech, Morrisville, NC, USA) and was then manually recorded.

### 2.5 Haemodynamics

$\dot{Q}$ was estimated non‐invasively using impedance cardiography. Six electrodes (PhysioFlow, HTFS‐50) were placed following the manufacturer's instructions for exercise testing: one on the sternum, one on the rib closest to V6, two on the left side of the neck, and two left and lateral to the midpoint of the spine. HR was determined from the electrocardiogram using the R‐R interval. The bioimpedance method uses changes in transthoracic impedance during cardiac ejection to calculate stroke volume and subsequently $\dot{Q}$ (Moshkovitz et al., 2004). The formula for the calculation of stroke volume and haemodynamic measurements is considered proprietary information by PhysioFlow, and as such is unknown. Impedance cardiography has proven to be an effective tool for evaluating $\dot{Q}$ and other haemodynamic variables during exercise (Charloux et al., 2000; Louvaris et al., 2019; Richard et al., 2001; Schultz et al., 2012; Valcicak et al., 2022). SVC was chosen over systemic vascular resistance as an index of vascular tone because greater relative changes were observed in $\dot{Q}$ than in MAP. As such, SVC provides a more linear measurement of vascular tone which is less dependent on the participant's starting point (Joyce et al., 2019).

### 2.6 Blood pressure

BP was measured non‐invasively via an automatic sphygmomanometer (Tango M2, SunTech) on the participant's dominant arm. An arm cuff was placed on the participant's upper arm, with care taken to ensure that the microphone was placed directly over the brachial artery, just proximal to the elbow. Three electrocardiogram leads were placed on the participant; one at V2, one at V6 and one on the lower right side of the participant's abdomen. Measurements were collected in Dimensional K‐sound Analysis mode to ensure accurate measurements during the exercise. MAP was calculated using systolic blood pressure (SBP) and diastolic blood pressure (DBP) using the equation:

$$\mathrm{MAP}=\frac{1}{3}\mathrm{SBP}+\frac{2}{3}\mathrm{DBP}$$

### 2.7 Statistical analysis

Statistical analyses were performed using SPSS Statistics 28.0.1.0 (IBM Corp., Armonk, NY, USA). Student's unpaired *t*‐test was used to assess differences in participant characteristics between groups, and Cohen's *d* was used to assess effect size (small, *d* = 0.2; medium, *d* = 0.5; and large, *d* ≥ 0.8). To examine the effects of drug treatment (Drug; saline, esmolol), exercise intensity (Intensity; rest, moderate exercise, heavy exercise), and age group (Age; younger, older) on physiological variables, the linear mixed model procedure was used. Age, Drug, Intensity, Age × Drug, Age × Intensity, Drug × Intensity, and Age × Drug × Intensity effects were modelled as fixed effects, and participant and absolute exercise power output were included as random factors. Covariance structure selection was based on optimizing the fit statistics based on the Bayesian information criterion. Statistical significance was set at *P* ≤ 0.05. When a significant main effect existed, the conservative Bonferroni correction method was used to adjust for multiple comparisons for all outcomes. Based on data from a previous study in our lab (Proctor et al., 2018), 20 subjects (10 younger, 10 older) provided 80% power to detect meaningful physiological differences in esmolol‐induced reduction of MAP (5 mmHg) between age groups during moderate intensity cycling exercise. Data are presented as means ± standard deviation (SD), unless otherwise specified.

To discern the relative influence of fitness on physiological variables, a subgroup analysis was performed within age groups (Table 2). The participants in each age group were divided into two fitness groups (FitGroup; high‐fit, low‐fit) based on the median absolute power output of the heavy‐intensity exercise bout. The age groups were analysed separately using the linear mixed model procedure. FitGroup, Drug, Intensity, FitGroup × Drug, FitGroup × Intensity, Drug × Intensity, and FitGroup × Drug × Intensity effects were modelled as fixed effects, and participant and absolute exercise power output were included as random factors. Covariance structure selection was based on optimizing the fit statistics based on the Bayesian information criterion. Statistical significance was set at *P* ≤ 0.05.

**TABLE 2:** Subgroup analysis.

	Heart rate		Cardiac output		MAP		SVC	
	Younger	Older	Younger	Older	Younger	Older	Younger	Older
FitGroup	0.693	0.518	0.922	0.335	0.703	0.514	0.958	0.632
Drug	<0.001	<0.001	<0.001	<0.001	0.049	<0.001	<0.001	0.152
Intensity	<0.001	<0.001	<0.001	<0.001	<0.001	<0.001	<0.001	<0.001
FitGroup × Drug	0.381	0.725	0.171	0.316	0.263	0.186	0.374	0.405
FitGroup × Intensity	0.844	0.398	0.329	0.985	0.832	0.505	0.773	0.688
Drug × Intensity	0.005	0.140	0.015	0.081	0.021	0.012	0.406	0.635
FitGroup × Drug × Intensity	0.795	0.992	0.968	0.961	0.396	0.330	0.246	0.982

*Note*: Data presented as *P*‐values. Drug: saline, esmolol; intensity: rest, moderate exercise, heavy exercise; FitGroup: low fitness, high fitness. Younger: *n* = 13; low fit, *n* = 7; high fit, *n* = 6; older: *n* = 14; low fit *n* = 7; high fit, *n* = 7.

## 3 RESULTS

### 3.1 Subject data

Twenty‐seven subjects (13 younger, 14 older) completed this study. Physical characteristics and peak exercise responses are provided in Table 1. These data reflect that the participants were healthy and that recent physical activity was not found to be significantly different between age groups (total activity, *P* = 0.093, Cohen's *d* = 0.780).

### 3.2 Cardiac responses

**FIGURE 3:**
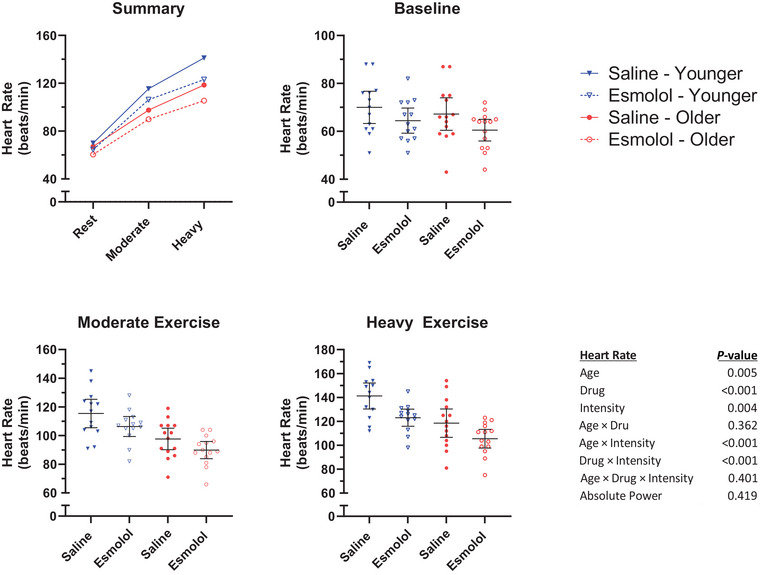
Heart rate. Mean HR during the final minute of each stage. Horizontal lines represent the group mean. Error bars represent the 95% confidence interval.

HR responses are presented in Figure 3 (13 younger, 14 older). A significant Drug × Intensity interaction was observed in HR (*P *< 0.001), with esmolol reducing HR more during heavy exercise (saline 130, esmolol 114, difference −16 (95% CI, −19 to −12) beats/min, *P *< 0.001) than during rest (saline 69, esmolol 62, difference −6 (95% CI, −9 to −3) beats/min, *P *< 0.001), or moderate exercise (saline 107, esmolol 98, difference −8 (95% CI, −12 to −5) beats/min, *P *< 0.001). Additionally, a significant Age × Intensity effect was observed in HR (*P *< 0.001), with younger women demonstrating higher HRs than older women during moderate (younger 111, older 94, difference +17 (95% CI, 7–27) beats/min, *P* = 0.001) and heavy (younger 132, older 112, difference +20 (95% CI, 10–30) beats/min, *P *< 0.001) exercise, but not during rest (younger 67, older 64, difference +3 (95% CI, −7 to 13) beats/min, *P* = 0.481). The Age × Drug (*P* = 0.362) and Age × Drug × Intensity (*P* = 0.401) interaction effects were not found to be significant.

**FIGURE 4:**
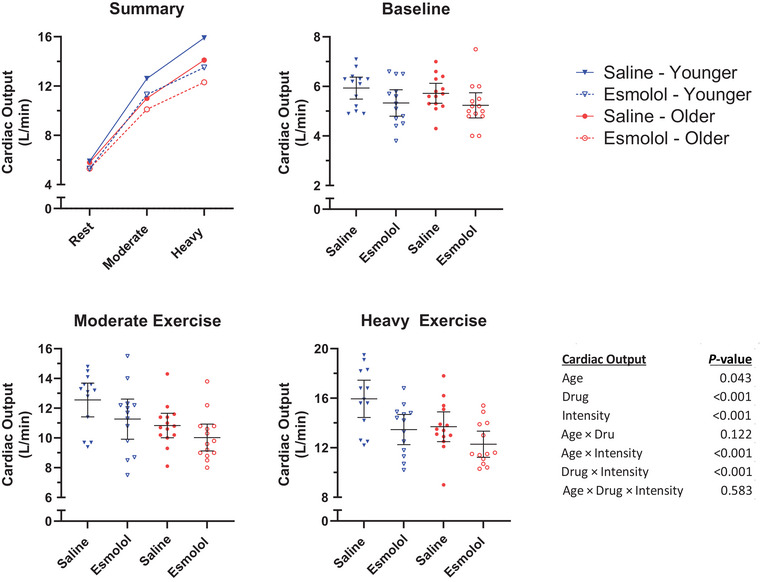
Cardiac output. Mean $\dot{Q}$ during the final minute of each stage. Horizontal lines represent the group mean. Error bars represent the 95% confidence interval.

$\dot{Q}$ responses are presented in Figure 4 (13 younger, 13 older). A significant Drug × Intensity interaction was observed in $\dot{Q}$ (*P* = 0.001), with esmolol reducing $\dot{Q}$ during moderate (saline 11.7, esmolol 10.6, difference −1.0 (95% CI, −1.6 to −0.5) L/min, *P *< 0.001) and heavy (saline 14.8, esmolol 12.8, difference −2.0 (95% CI, −2.6 to −1.5) L/min, *P *< 0.001) exercise, but not during rest (saline 5.8, esmolol 5.3, difference −0.5 (95% CI, −1.1 to 0.0) L/min, *P* = 0.048). Additionally, a significant Age × Intensity effect was observed in $\dot{Q}$ (*P *< 0.001) with younger women demonstrating higher $\dot{Q}$ than older women during moderate (younger 11.9, older 10.4, difference +1.5 (95% CI, 0.3–2.7) L/min, *P* = 0.015) and heavy (younger 14.7, older 12.9, difference +1.8 (95% CI, 0.6–3.0) L/min, *P* = 0.004) exercise, but not during rest (younger 5.6, older 5.5, difference +0.2 (95% CI, −1.0 to 1.3) beats/min, *P* = 0.794). The Age × Drug (*P* = 0.122) and Age × Drug × Intensity (*P* = 0.583) interaction effects were not found to be significant.

### 3.3 Blood pressure

**FIGURE 5:**
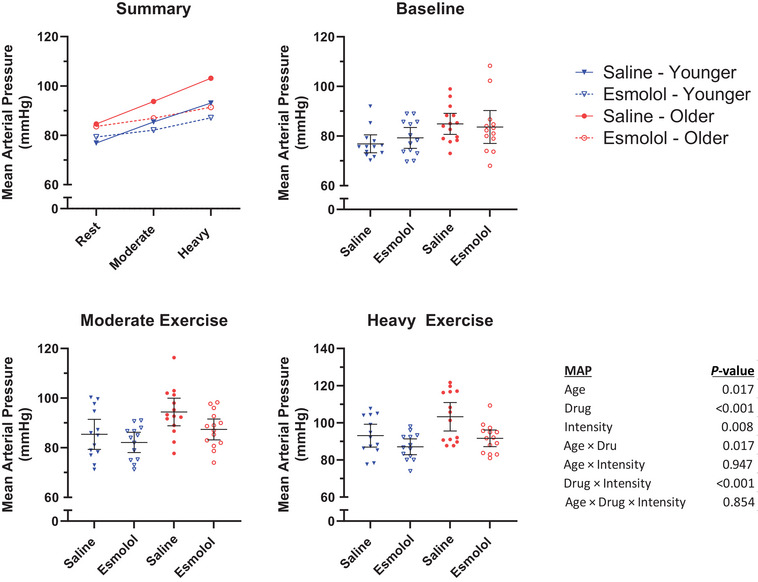
Mean arterial pressure. Mean MAP during the final minute of each stage. Horizontal lines represent the group mean. Error bars represent the 95% confidence interval.

MAP responses are presented in Figure 5 (13 younger, 13 older). A significant Age × Drug interaction was observed in MAP (*P* = 0.017), with esmolol significantly reducing MAP in the older group (saline 94, esmolol 88, difference −7 (95% CI, −9 to −4) mmHg, *P *< 0.001), but not in the younger group (saline 85, esmolol 83, difference −2 mmHg (95% CI, −5 to 0) mmHg, *P* = 0.071). A significant Drug × Intensity effect was also observed in MAP (*P *< 0.001), with esmolol reducing MAP during moderate (saline 90, esmolol 85, difference −5 (95% CI, −8 to –2) mmHg, *P* = 0.001) and heavy (saline 98, esmolol 89, difference −9 (95% CI, −12 to −6) mmHg, *P *< 0.001) exercise, but it was not reduced at rest (saline 81, esmolol 82, difference +1 (95% CI, −2 to 4) mmHg, *P* = 0.637). The Age × Intensity (*P* = 0.947) and Age × Drug × Intensity (*P* = 0.854) interaction effects were not found to be significant.

### 3.4 Systemic vascular conductance

**FIGURE 6:**
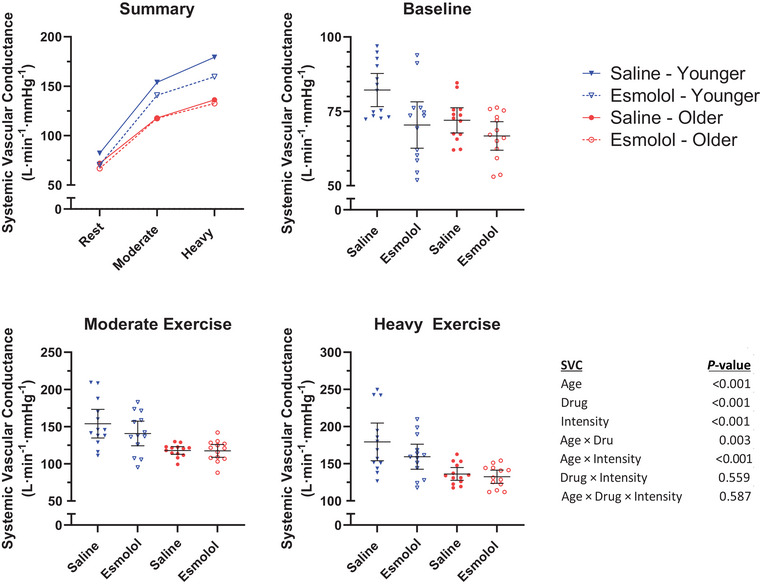
Systemic vascular conductance. Mean SVC during the final minute of each stage. Horizontal lines represent the group mean. Error bars represent the 95% confidence interval.

SVC responses are presented in Figure 6 (13 younger, 13 older). A significant Age × Drug interaction was observed in SVC (*P* = 0.003), with esmolol significantly reducing SVC in younger women (saline 138, esmolol 123, difference −15 (95% CI, −20 to −10) mL/min/mmHg, *P *< 0.001), but not older women (saline 109, esmolol 106, difference −3 (95% CI, −9 to 2) mL/min/mmHg, *P* = 0.209). Additionally, a significant Age × Intensity interaction was also observed (*P *< 0.001), with SVC being significantly lower in older women during moderate (younger 147, older 117, difference −30 (95% CI, −44 to −15) mL/min/mmHg, *P *< 0.001) and heavy (younger 169, older 134, difference −35 (95% CI, −50 to −20) mL/min/mmHg, *P *< 0.001) exercise, but not during rest (younger 76, older 69, difference −7 (95% CI, −20 to 6) mL/min/mmHg, *P* = 0.285). The Drug × Intensity (*P* = 0.559) and Age × Drug × Intensity (*P* = 0.587) interaction effects were not found to be significant.

### 3.5 Sensitivity analysis

Results of the sensitivity analysis are presented in Table 2. There were no significant effects of FitGroup, FitGroup × Drug, FitGroup × Intensity, or FitGroup × Drug × Intensity in either age group for any of the measured outcomes. Conversely, Intensity and Drug were significant for every outcome measure except SVC in the older group.

## 4 DISCUSSION

The major findings of this study reveal significant age‐related differences in vascular BP regulation in women. Specifically, we found that older, postmenopausal women have a diminished ability to maintain MAP by reducing SVC when $\dot{Q}$ is attenuated by cardioselective β‐blockade compared to younger women.

Evidence suggests that older women may have difficulty adequately adjusting SVC to maintain BP (Credeur et al., 2014; Trinity et al., 2018). However, previous studies assessing this during exercise utilized primarily small muscle models (Choi et al., 2012; Fadel et al., 2004; Grotle et al., 2023; Kang et al., 2022; Petrofsky & Lind, 1975; Ranadive et al., 2017; Trinity et al., 2018; Watso et al., 2020; Wenner et al., 2022). This study utilized acute cardioselective β‐blockade to simultaneously instigate a hypotensive challenge as well as attenuate the central cardiac response to such a stimulus in order to assess limitations in the peripheral vascular response. The central questions of this study were: (1) Will older women have a greater reduction in MAP during cardioselective β‐blockade compared to younger women? and (2) Will older women exhibit a smaller reduction in SVC in response to cardioselective β‐blockade relative to younger women? Consistent with our hypotheses, older women demonstrated a reduced ability to maintain MAP when $\dot{Q}$ was limited by cardioselective β‐blockade relative to their younger counterparts. Additionally, older women exhibited a smaller reduction in SVC in response to cardioselective β‐blockade relative to younger women despite similar reductions in $\dot{Q}$.

### 4.1 Cardiac responses

As expected, HR and $\dot{Q}$ were both reduced by esmolol. Fat‐free mass, rather than total body mass, was used because fat‐free mass is correlated with total blood volume (Hunt et al., 1998) and esmolol is metabolized by red blood cell esterases (Wiest & Haney, 2012). Thus, dosing based on fat free mass accounts for differences in body composition, which is of particular importance when making comparisons between age groups. In addition, the lack of a significant Age × Drug or Age × Drug × Intensity interaction with respect to HR (Drug × Age, *P* = 0.362; Age × Drug × Intensity, *P* = 0.401) or $\dot{Q}$ (Age × Drug, *P* = 0.112; Age × Drug × Intensity, *P* = 0.583) suggests that drug sensitivity was reasonably similar between age groups. Therefore, changes in downstream peripheral vascular variables likely reflect structural or functional changes between groups and not a difference in drug sensitivity.

### 4.2 Vascular blood pressure regulation during cardioselective β‐blockade

The present study identifies significant age differences in vascular BP regulation in women. Compared to the younger women, the older women exhibited a greater reduction in MAP when $\dot{Q}$ was attenuated by acute, cardioselective β‐blockade. Additionally, younger women significantly lowered SVC during esmolol infusion, but older women did not despite similar reductions in $\dot{Q}$. Together these findings demonstrate that older women have a reduced ability to maintain MAP via systemic vasoconstriction relative to younger women both at rest and during large muscle mass exercise.

In a previous study by Credeur et al. (2014), positive neck pressure was used to simulate hypotensive activation of the carotid baroreceptor in resting younger and older women. In response, older women exhibited a blunted ability to increase MAP to hypotensive stimuli. While both groups demonstrated similar reductions in conductance, older women displayed a much smaller increase in $\dot{Q}$. This suggests that (1) the blunted ability to increase MAP during a hypotensive stimulus in older women is driven by an attenuated cardiac response and (2) older women are unable to sufficiently lower conductance to overcome this attenuated $\dot{Q}$ response.

The present study expands upon these findings. Cardioselective β‐blockade was utilized to both instigate a hypotensive stimulus and also to attenuate the ability to increase $\dot{Q}$ in response to such a stimulus. Despite previous observations demonstrating an increased reliance on this cardiac response (Credeur et al., 2014; Kim et al., 2011), younger women showed a greater ability to compensate by reducing SVC significantly more than older women during cardioselective β‐blockade, resulting in a smaller reduction in MAP. This suggests that not only is the ability of older women to lower conductance insufficient to overcome the deficit in $\dot{Q}$, but it is also impaired relative to younger women. Moreover, the present study included exercise extending these findings beyond the resting condition. This is of particular relevance as, in addition to the baroreflex, exercise also stimulates vasoconstriction via other mechanisms including central command and the exercise pressor reflex. Last, the study by Credeur et al. (2014) examined acute, beat‐to‐beat BP regulation during brief 10 s time periods (5 s of neck pressure and 5 s after pressure release). In the present study, we observed that these deficits in systemic vasoconstriction are persistent and represent prolonged deficits in BP regulation. It is worth noting that, in addition to sympathetic vasoconstriction of inactive tissues, exercise also induces vasodilatation in active muscles. As such, the present findings may represent a difference in the integration of these BP responses, rather than a reduction in the ability to vasoconstrict. However, the observed age difference in SVC in response to esmolol infusion was not observed to be significantly altered by exercise (Age × Drug × Intensity, *P* = 0.587).

### 4.3 Age and exercise

In a previous study by Trinity et al. (2018), younger (18–30 years) and older (>65 years) men and women performed dynamic plantar flexion exercises at 40% of their maximal plantar flexion work rate. They found that the older women had an exaggerated increase in MAP in response to plantar flexion exercise. The authors attributed this exaggerated BP response to an inability to decrease systemic vascular resistance (the inverse of SVC), unlike men and younger women. In the present study, the older women did increase SVC in response to exercise; however, this increase was attenuated relative to the younger women (Age × Intensity, *P* = 0.003). This inability to increase SVC during exercise may contribute to the reduced ability to increase $\dot{Q}$ observed in older women (Age × Intensity, *P *< 0.001). Additionally, despite older women exhibiting an increased MAP relative to younger women (Age × Drug, *P* = 0.017), the exercise‐induced rise in MAP was not different between the age groups (Age × Intensity, *P* = 0.947). Together, these findings demonstrate that older women have an impaired ability to increase SVC during exercise. However, this impairment appears to manifest differently between small and large muscle mass exercises. Trinity et al. (2018) also noted that popliteal vascular conductance was not different between younger and older men and women, and used this evidence to propose that the impaired ability to reduce systemic vascular resistance in older women is driven by greater sympathetic vasoconstriction in the inactive vasculature of older women, rather than impaired active muscle vasodilatation. If this hypothesis is correct, then the attenuated, but still elevated, SVC observed in older women in the present study likely results from a larger active muscle mass compared to the calf model used by Trinity et al. However, as the present study did not measure active leg conductance this cannot be confirmed.

### 4.4 Effects of fitness and physical activity

One important consideration for the present study is the difference in fitness between age groups, as evidenced by the significantly lower ${\dot{V}}_{O_{2}\mathrm{peak}}$ and absolute workloads observed in the older women (Table 1). However, as fitness naturally declines with age, attempting to match age groups for fitness would likely require matching sedentary younger women with very active older women. To further investigate the effects of fitness, a subgroup analysis was performed (Table 2). FitGroup explained less variation than Age and was not significant for any of the measured outcomes in either age group. This suggests that varying fitness between age groups alone is not sufficient to explain any of the observed differences and that additional age‐related factors must play at least some role.

We did not observe a significant difference in self‐reported physical activity between groups. However, given the low *P*‐value and medium‐to‐large effect size (total activity, *P* = 0.093, Cohen's *d* = 0.780), this may reflect that the study is underpowered to adequately detect a difference in physical activity. It is worth noting, however, that total activity was determined using a self‐report questionnaire. As such, participants were estimating their physical activity rather than tracking it. Last, the questionnaire only asks about physical activity performed over the previous week, which may or may not be indicative of the participants’ habitual activity level.

### 4.5 Perspectives

The present findings have important implications for future research into age‐related changes in systemic BP regulation and vascular control in women. First, this is the third study to utilize esmolol infusion during exercise (Muller et al., 2017; Proctor et al., 2018), further demonstrating the safety and utility of infusion of esmolol, which due to its rapid half‐life (<9 min) enables randomized, within‐visit comparisons of saline versus pharmacological blockade, for investigating cardiovascular function. Additionally, the blunted ability to respond to hypotensive challenges in older women may have significant clinical implications, particularly during hypotensive stressors such as heat stress, hypovolaemia and orthostatic hypotension.

This study highlights the need for more integrative research into the age‐related changes in vascular responsiveness in women, particularly during large muscle exercise. While the present study does identify vascular limitations in BP regulation of older women, the underlying mechanism(s) remain uncertain. Likely contributors include arterial stiffening and the loss of β_2_‐adrenergic vasodilatation. Arterial stiffening has been associated with diminished baroreceptor sensitivity (Okada et al., 2012), impaired vasodilatation (Rodrigo et al., 2011) and reduced vasoconstriction (Kang et al., 2009; Muller‐Delp et al., 2002). β_2_‐Mediated adrenergic vasodilatation has been observed to offset α_1_‐mediated vasoconstriction in younger, but not older, women (Hart et al., 2011). Indeed, loss of β_2_ vasodilatation may underlie the lower SVC and higher MAP observed in older women in this study. It is also possible that older women are operating closer to their maximal vasoconstrictor capacity in inactive tissues, inhibiting their ability to adjust to hypotensive stimuli. Another area warranting additional research is the role of menopause and/or sex hormones on these age‐related vascular changes in women.

### 4.6 Experimental considerations

While the present study was able to identify vascular limitations in older women, it is unable to determine if these limitations in BP regulation are further exacerbated during exercise (Age × Drug × Intensity, *P* = 0.587). However, some evidence from the current study does suggest that this may be the case. As previously established, esmolol significantly reduced MAP more in older women than in the younger women (*P *< 0.001), and esmolol only significantly reduced MAP during exercise (moderate, *P* = 0.002; heavy, *P *< 0.001), but not during rest (*P* = 0.637). Together these findings suggest that exercise may exacerbate the age‐related deficits in BP regulation in older women.

While our measurement techniques allowed for a non‐invasive and integrative assessment, few would be considered direct or ‘gold‐standard’ measurements. Additionally, given that measurements of BP are already used to estimate $\dot{Q}$ using impedance cardiography, BP may be ‘double‐counted’ when calculating SVC using Ohm's Law. Future studies hoping to investigate any underlying mechanisms would benefit from using more direct measurements, such as echocardiography and Doppler ultrasound.

### 4.7 Summary

In summary, utilizing cardioselective β‐blockade and large muscle mass exercise, we revealed a limitation in the peripheral vasoconstrictor capacity of older, postmenopausal women. Despite the similar reduction in $\dot{Q}$, older women experienced a greater reduction in MAP and a smaller decline in SVC relative to younger women. Taken together, this implies that despite their increased reliance on altering SVC to compensate for diminished cardiac reserve, the ability of systemic vasoconstriction to maintain BP in older women is actually diminished relative to their younger counterparts.

## AUTHOR CONTRIBUTIONS

David N. Proctor, Matthew J. Studinski, Matthew D. Muller, and Swapan Mookerjee conceived and designed the study. Matthew J. Studinski, Christine Bowlus, Swapan Mookerjee, Jason Fragin, Jigar Gosalia, and Jocelyn M. Delgado Spicuzza performed the experiments and assisted with data collection. Matthew J. Studinski and David N. Proctor analysed data. Matthew J. Studinski, David N. Proctor, James A. Pawelczyk, and Matthew D. Muller interpreted the results. Matthew J. Studinski drafted the manuscript. David N. Proctor, Christine Bowlus, James A. Pawelczyk, Matthew D. Muller, Swapan Mookerjee, Jason Fragin, Jocelyn M. Delgado Spicuzza, and Jigar Gosalia edited and revised the manuscript. All authors have read and approved the final version of this manuscript and agree to be accountable for all aspects of the work in ensuring that questions related to the accuracy or integrity of any part of the work are appropriately investigated and resolved. All persons designated as authors qualify for authorship, and all those who qualify for authorship are listed.

## CONFLICT OF INTEREST

None declared.

## Data Availability

The data that supports the findings of this study are available in the supplementary material of this article.
